# Methyl 2-(5-fluoro-1*H*-indol-3-yl)-2-oxoacetate

**DOI:** 10.1107/S1600536809053033

**Published:** 2009-12-16

**Authors:** Shuping Wang, Huajiang Dong, Hong Chen, Kunpeng Zhu, Tieliang Zhu

**Affiliations:** a The Affiliated Hospital of the Medical College of the Chinese People’s Armed Police Forces, Tianjin 300162, People’s Republic of China

## Abstract

The indolyl portion of the title mol­ecule, C_11_H_8_FNO_3_, is flat, the five- and six-membered rings making a dihedral angle of 0.815 (6)°. Inter­molecular N—H⋯O hydrogen bonds link adjacent mol­ecules into a linear chain. Slipped π–π stacking inter­actions between two neighboring indole groups further consolidate the mol­ecules into a three-dimensional supra­molecular architecture [centroid–centroid distances = 3.555 (10) and 3.569 (10) Å].

## Related literature

For the biological activity of the title compound and its derivatives, see: Kozikowski *et al.* (2006 ▶); Albert *et al.* (2002 ▶); Jaquith *et al.* (2005 ▶). For the preparation, see: Alawar *et al.* (2004 ▶).
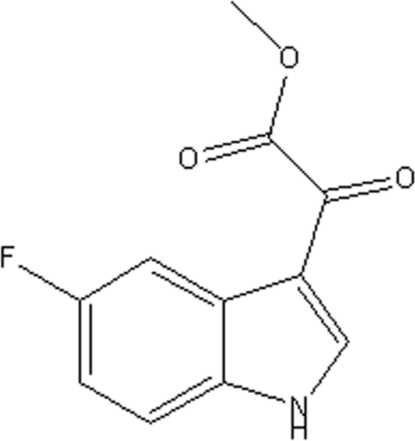


## Experimental

### 

#### Crystal data

C_11_H_8_FNO_3_
*M* *_r_* = 221.18
Monoclinic, 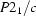

*a* = 7.0584 (14) Å
*b* = 20.586 (4) Å
*c* = 7.3286 (15) Å
β = 112.01 (3)°
*V* = 987.3 (3) Å^3^
*Z* = 4
Mo *K*α radiation
μ = 0.12 mm^−1^
*T* = 113 K
0.30 × 0.24 × 0.20 mm

#### Data collection

Rigaku Saturn CCD area-detector diffractometer
Absorption correction: multi-scan (*CrystalClear*; Rigaku/MSC, 2004 ▶) *T*_min_ = 0.964, *T*_max_ = 0.976
9472 measured reflections
2328 independent reflections
2097 reflections with *I* > 2σ(*I*)
*R*_int_ = 0.024

#### Refinement

*R*[*F* ^2^ > 2σ(*F* ^2^)] = 0.037
*wR*(*F* ^2^) = 0.097
*S* = 1.06
2328 reflections
150 parameters
H atoms treated by a mixture of independent and constrained refinement
Δρ_max_ = 0.38 e Å^−3^
Δρ_min_ = −0.20 e Å^−3^

### 

Data collection: *CrystalClear* (Rigaku/MSC, 2004 ▶); cell refinement: *CrystalClear*; data reduction: *CrystalClear*; program(s) used to solve structure: *SHELXL97* (Sheldrick, 2008 ▶); program(s) used to refine structure: *SHELXL97* (Sheldrick, 2008 ▶); molecular graphics: *DIAMOND* (Brandenburg, 1999 ▶); software used to prepare material for publication: *publCIF* (Westrip, 2009 ▶).

## Supplementary Material

Crystal structure: contains datablocks global, I. DOI: 10.1107/S1600536809053033/ng2701sup1.cif

Structure factors: contains datablocks I. DOI: 10.1107/S1600536809053033/ng2701Isup2.hkl

Additional supplementary materials:  crystallographic information; 3D view; checkCIF report

## Figures and Tables

**Table 1:** Hydrogen-bond geometry (Å, °)

*D*—H⋯*A*	*D*—H	H⋯*A*	*D*⋯*A*	*D*—H⋯*A*
N1—H1⋯O3^i^	0.932 (17)	2.420 (16)	3.1598 (13)	136.3 (13)
N1—H1⋯O1^i^	0.932 (17)	1.932 (17)	2.7861 (13)	151.3 (14)

Symmetry code: (i) 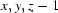.

**Fig. 1.**
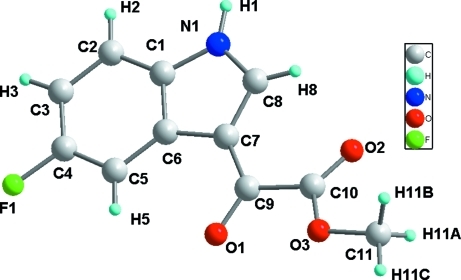
The molecular structure and atom-labeling scheme of (I).

**Fig. 2.**
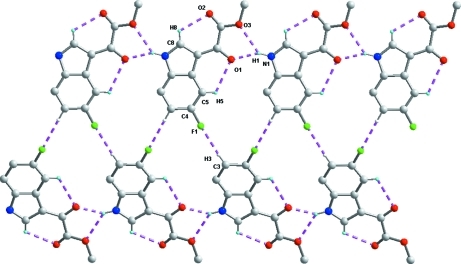
The corresponding N—H···O, C—H···O and C—H···F hydrogen bonds stabling the packing structure of (I). Hydrogen bonds are shown as dashed lines.

**Fig. 3.**
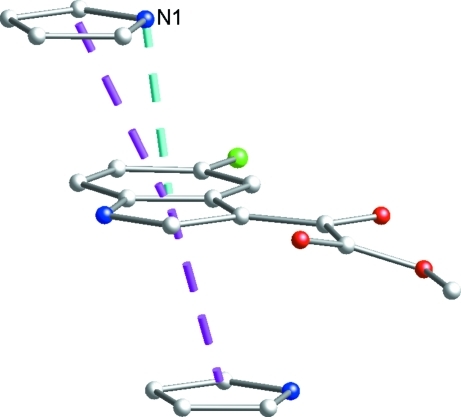
The π-π stacking interactions and N—H···π interactions in (I). Hydrogen bonds are shown as dashed lines.

**Fig. 4.**
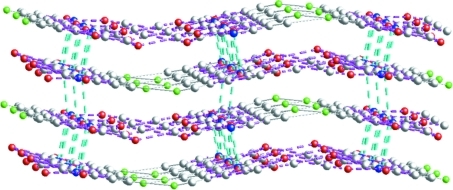
The three-dimensional supramolecular packing architecture of (I). cyan represent π-π stacking interactions and N—H···π interactions while purple represent N—H···O, C—H···F and C—H···O interactions.

## supplementary crystallographic information

### Comment

The synthesis of the title compound (I) and its derivatives has received considerable attention on account of their biological activity especially for both enzymic and cell-based study (Kozikowski *et al.*, 2006). They also can be used as neuroprotective (Albert *et al.*, 2002) and anti-proliferative agents (Jaquith *et al.*, 2005).

In this work, the title molecule, C_11_H_8_FNO_3_, (Fig. 1), has been synthesized. In an asymmetric unit of (I) one molecule can be observed. (I) crystalizes in the Monoclinic P2(1)/n space group, a = 7.0584 (14) Å, b = 20.586 (4) Å, c = 7.3286 (15) Å, β = 112.01 (3)°. The heterocyclic rings (N1, C1, C6, C7, C8) make a small dihedral angle of 0.815 (6)° with benzene rings (C1, C2, C3, C4, C5, C6,). N atoms in the molecule act as hydrogen-bond donors to O atoms in the neighbouring molecule forming intermolecular N1—H1···O1 (symmetry code: *x*, *y*, *z* - 1) and N1—H1···O3 (symmetry code: *x*, *y*, *z* - 1) hydrogen bonds. These N—H···O hydrogen bonds, C3—H3···F1(symmetry code: *x*, -*y* + 1/2, *z* - 1/2), and intra-molecular C5—H5···O1, C8—H8···O2 hydrogen bonds stabilize the crystal structure and extend molecules (I) into a double-tape structure along the c direction. π–π interactions between the indole rings are also present, the centroid-centroid distances [symmetry code: 1 - *x*,-*y*,2 - *z*; 2 - *x*,-*y*,2 - *z*] are 3.555 (10) and 3.569 (10) Å. N—H···π interactions [symmetry code: 1 - *x*,-*y*,2 - *z*] are 3.218 (21) Å. These Parallel slipped π-π stacking interactions between two neighboring indole groups and N—H···π interactions also further consolidate (I) into the three-dimensional supramolecular architecture.

Perspective drawing with the atomic numbering scheme is illustrated in Figure 1. Selected geometric parameters (Å, °) for (I) are listed in table 1. Selected hydrogen-bonding geometric parameters (Å, °) for (I) are listed in table 2. The corresponding N—H···O, C—H···O and C—H···F hydrogen bonds are shown in Figure 2. The π-π stacking interactions and N—H···π interactions are shown in Ffigure 3. The three-dimensional supramolecular packing architecture of (I) is shown in Figure 4.

### Experimental

Methyl 2-(5-fluoro-1*H*-indol-3-yl)-2-oxoacetate was prepared according to the previous literature (Alawar *et al.*, 2004). Colorless block Single crystals were obtained by slow evaporation from a methanol and water mixed solution (150 ml) of (I) at room temperature.

### Refinement

H atoms on N atoms were located in a difference Fourier map and refined isotropically, with restrains of N—H = 0.90±0.01 Å. Other H atoms were positioned geometrically with C—H = 0.93 and 0.96 Å, for indole H atoms, respectively, and refined using a riding model, with *U*_iso_(H) =1.2Ueq(C)(1.5 for methyl groups).

### Crystal data

**Table:**

C_11_H_8_FNO_3_	*F*(000) = 456
*M**_r_* = 221.18	*D*_x_ = 1.488 Mg m^−^^3^
Monoclinic, *P*2_1_/*c*	Mo *K*α radiation, λ = 0.71073 Å
Hall symbol: -P 2ybc	Cell parameters from 2958 reflections
*a* = 7.0584 (14) Å	θ = 3.1–27.9°
*b* = 20.586 (4) Å	µ = 0.12 mm^−^^1^
*c* = 7.3286 (15) Å	*T* = 113 K
β = 112.01 (3)°	Block, colorless
*V* = 987.3 (3) Å^3^	0.30 × 0.24 × 0.20 mm
*Z* = 4	

### Data collection

**Table:**

Rigaku Saturn CCD area-detector diffractometer	2328 independent reflections
Radiation source: rotating anode	2097 reflections with *I* > 2σ(*I*)
multilayer	*R*_int_ = 0.024
Detector resolution: 7.31 pixels mm^-1^	θ_max_ = 27.9°, θ_min_ = 3.1°
ω and φ scans	*h* = −9→8
Absorption correction: multi-scan (*CrystalClear*; Rigaku/MSC, 2004)	*k* = −27→26
*T*_min_ = 0.964, *T*_max_ = 0.976	*l* = −9→9
9472 measured reflections	

### Refinement

**Table:**

Refinement on *F*^2^	0 restraints
Least-squares matrix: full	H atoms treated by a mixture of independent and constrained refinement
*R*[*F*^2^ > 2σ(*F*^2^)] = 0.037	*w* = 1/[σ^2^(*F*_o_^2^) + (0.0503*P*)^2^ + 0.338*P*] where *P* = (*F*_o_^2^ + 2*F*_c_^2^)/3
*wR*(*F*^2^) = 0.097	(Δ/σ)_max_ = 0.001
*S* = 1.06	Δρ_max_ = 0.38 e Å^−^^3^
2328 reflections	Δρ_min_ = −0.20 e Å^−^^3^
150 parameters	

### Special details

**Table:**

Geometry. All e.s.d.'s (except the e.s.d. in the dihedral angle between two l.s. planes) are estimated using the full covariance matrix. The cell e.s.d.'s are taken into account individually in the estimation of e.s.d.'s in distances, angles and torsion angles; correlations between e.s.d.'s in cell parameters are only used when they are defined by crystal symmetry. An approximate (isotropic) treatment of cell e.s.d.'s is used for estimating e.s.d.'s involving l.s. planes.
Refinement. Refinement of *F*^2^ against ALL reflections. The weighted *R*-factor *wR* and goodness of fit *S* are based on *F*^2^, conventional *R*-factors *R* are based on *F*, with *F* set to zero for negative *F*^2^. The threshold expression of *F*^2^ > σ(*F*^2^) is used only for calculating *R*-factors(gt) *etc*. and is not relevant to the choice of reflections for refinement. *R*-factors based on *F*^2^ are statistically about twice as large as those based on *F*, and *R*- factors based on ALL data will be even larger.

### Fractional atomic coordinates and isotropic or equivalent isotropic displacement parameters (Å^2^)

**Table:**

	*x*	*y*	*z*	*U*_iso_*/*U*_eq_	
F1	0.12543 (14)	0.29293 (3)	0.65340 (12)	0.0361 (2)	
O1	0.26544 (13)	0.51327 (4)	0.99608 (11)	0.02011 (19)	
O2	0.38836 (16)	0.66111 (4)	0.83866 (13)	0.0297 (2)	
N1	0.26610 (14)	0.52509 (5)	0.37504 (13)	0.0173 (2)	
O3	0.28315 (14)	0.63607 (4)	1.08373 (12)	0.0244 (2)	
C1	0.23060 (16)	0.46201 (5)	0.42115 (15)	0.0162 (2)	
C2	0.19687 (18)	0.40616 (6)	0.30676 (16)	0.0215 (2)	
H2	0.1971	0.4072	0.1772	0.026*	
C3	0.16302 (19)	0.34900 (6)	0.38896 (18)	0.0247 (3)	
H3	0.1396	0.3095	0.3167	0.030*	
C4	0.16368 (19)	0.35010 (5)	0.57927 (18)	0.0232 (3)	
C5	0.20002 (17)	0.40431 (5)	0.69689 (16)	0.0187 (2)	
H5	0.2012	0.4025	0.8269	0.022*	
C6	0.23508 (16)	0.46224 (5)	0.61507 (15)	0.0149 (2)	
C7	0.27633 (15)	0.52844 (5)	0.68412 (15)	0.0146 (2)	
C8	0.29297 (16)	0.56415 (5)	0.52914 (15)	0.0161 (2)	
H8	0.3194	0.6095	0.5323	0.019*	
C9	0.28704 (16)	0.55005 (5)	0.87314 (15)	0.0151 (2)	
C10	0.32621 (17)	0.62255 (5)	0.92560 (15)	0.0183 (2)	
C11	0.3146 (2)	0.70317 (6)	1.14936 (19)	0.0327 (3)	
H11A	0.4534	0.7166	1.1666	0.049*	
H11B	0.2962	0.7072	1.2749	0.049*	
H11C	0.2154	0.7310	1.0508	0.049*	
H1	0.271 (2)	0.5363 (8)	0.253 (2)	0.036 (4)*	

### Atomic displacement parameters (Å^2^)

**Table:**

	*U*^11^	*U*^22^	*U*^33^	*U*^12^	*U*^13^	*U*^23^
F1	0.0544 (5)	0.0165 (3)	0.0358 (4)	−0.0065 (3)	0.0150 (4)	0.0025 (3)
O1	0.0294 (4)	0.0200 (4)	0.0134 (4)	−0.0006 (3)	0.0109 (3)	0.0013 (3)
O2	0.0479 (6)	0.0199 (4)	0.0264 (5)	−0.0071 (4)	0.0198 (4)	−0.0019 (3)
N1	0.0195 (5)	0.0220 (5)	0.0121 (4)	0.0002 (3)	0.0079 (4)	0.0007 (3)
O3	0.0397 (5)	0.0183 (4)	0.0178 (4)	0.0013 (3)	0.0141 (4)	−0.0039 (3)
C1	0.0150 (5)	0.0207 (5)	0.0134 (5)	0.0013 (4)	0.0058 (4)	−0.0006 (4)
C2	0.0218 (6)	0.0264 (6)	0.0171 (5)	0.0010 (4)	0.0082 (4)	−0.0057 (4)
C3	0.0263 (6)	0.0214 (5)	0.0247 (6)	0.0004 (4)	0.0077 (5)	−0.0078 (4)
C4	0.0266 (6)	0.0162 (5)	0.0254 (6)	−0.0003 (4)	0.0080 (5)	0.0022 (4)
C5	0.0209 (5)	0.0189 (5)	0.0155 (5)	0.0007 (4)	0.0061 (4)	0.0017 (4)
C6	0.0136 (5)	0.0184 (5)	0.0126 (5)	0.0012 (4)	0.0047 (4)	−0.0003 (4)
C7	0.0149 (5)	0.0174 (5)	0.0120 (5)	0.0011 (4)	0.0056 (4)	0.0009 (4)
C8	0.0167 (5)	0.0191 (5)	0.0130 (5)	0.0002 (4)	0.0061 (4)	0.0010 (4)
C9	0.0161 (5)	0.0170 (5)	0.0125 (5)	0.0010 (4)	0.0056 (4)	0.0000 (4)
C10	0.0219 (5)	0.0190 (5)	0.0130 (5)	0.0017 (4)	0.0054 (4)	−0.0007 (4)
C11	0.0547 (9)	0.0191 (6)	0.0251 (6)	0.0038 (5)	0.0158 (6)	−0.0059 (5)

### Geometric parameters (Å, °)

**Table:**

F1—C4	1.3650 (13)	C3—H3	0.9500
O1—C9	1.2295 (13)	C4—C5	1.3742 (16)
O2—C10	1.1992 (14)	C5—C6	1.3978 (14)
N1—C8	1.3406 (13)	C5—H5	0.9500
N1—C1	1.3881 (14)	C6—C7	1.4450 (14)
N1—H1	0.932 (17)	C7—C8	1.3943 (14)
O3—C10	1.3330 (13)	C7—C9	1.4297 (14)
O3—C11	1.4520 (14)	C8—H8	0.9500
C1—C2	1.3898 (15)	C9—C10	1.5400 (15)
C1—C6	1.4097 (14)	C11—H11A	0.9800
C2—C3	1.3829 (17)	C11—H11B	0.9800
C2—H2	0.9500	C11—H11C	0.9800
C3—C4	1.3932 (17)		
			
C8—N1—C1	109.72 (9)	C5—C6—C7	134.36 (9)
C8—N1—H1	127.7 (10)	C1—C6—C7	106.36 (9)
C1—N1—H1	122.6 (10)	C8—C7—C9	129.44 (10)
C10—O3—C11	115.49 (9)	C8—C7—C6	106.22 (9)
N1—C1—C2	129.26 (10)	C9—C7—C6	124.29 (9)
N1—C1—C6	107.72 (9)	N1—C8—C7	109.97 (9)
C2—C1—C6	123.02 (10)	N1—C8—H8	125.0
C3—C2—C1	117.37 (10)	C7—C8—H8	125.0
C3—C2—H2	121.3	O1—C9—C7	122.84 (10)
C1—C2—H2	121.3	O1—C9—C10	118.29 (9)
C2—C3—C4	119.07 (10)	C7—C9—C10	118.87 (9)
C2—C3—H3	120.5	O2—C10—O3	124.87 (10)
C4—C3—H3	120.5	O2—C10—C9	125.17 (10)
F1—C4—C5	117.96 (11)	O3—C10—C9	109.96 (9)
F1—C4—C3	117.22 (10)	O3—C11—H11A	109.5
C5—C4—C3	124.82 (11)	O3—C11—H11B	109.5
C4—C5—C6	116.43 (10)	H11A—C11—H11B	109.5
C4—C5—H5	121.8	O3—C11—H11C	109.5
C6—C5—H5	121.8	H11A—C11—H11C	109.5
C5—C6—C1	119.27 (9)	H11B—C11—H11C	109.5
			
C8—N1—C1—C2	−179.36 (11)	C1—C6—C7—C8	0.36 (11)
C8—N1—C1—C6	0.23 (12)	C5—C6—C7—C9	−1.23 (19)
N1—C1—C2—C3	−179.31 (11)	C1—C6—C7—C9	177.96 (10)
C6—C1—C2—C3	1.15 (17)	C1—N1—C8—C7	0.00 (12)
C1—C2—C3—C4	0.19 (17)	C9—C7—C8—N1	−177.67 (10)
C2—C3—C4—F1	178.67 (11)	C6—C7—C8—N1	−0.23 (12)
C2—C3—C4—C5	−1.38 (19)	C8—C7—C9—O1	178.43 (11)
F1—C4—C5—C6	−178.92 (10)	C6—C7—C9—O1	1.41 (17)
C3—C4—C5—C6	1.13 (18)	C8—C7—C9—C10	−1.25 (17)
C4—C5—C6—C1	0.25 (15)	C6—C7—C9—C10	−178.28 (10)
C4—C5—C6—C7	179.36 (12)	C11—O3—C10—O2	0.50 (17)
N1—C1—C6—C5	178.98 (10)	C11—O3—C10—C9	179.76 (10)
C2—C1—C6—C5	−1.40 (16)	O1—C9—C10—O2	164.82 (12)
N1—C1—C6—C7	−0.36 (11)	C7—C9—C10—O2	−15.49 (17)
C2—C1—C6—C7	179.26 (10)	O1—C9—C10—O3	−14.44 (14)
C5—C6—C7—C8	−178.83 (12)	C7—C9—C10—O3	165.25 (9)

### Hydrogen-bond geometry (Å, °)

**Table:**

*D*—H···*A*	*D*—H	H···*A*	*D*···*A*	*D*—H···*A*
N1—H1···O3^i^	0.932 (17)	2.420 (16)	3.1598 (13)	136.3 (13)
N1—H1···O1^i^	0.932 (17)	1.932 (17)	2.7861 (13)	151.3 (14)
C3—H3···F1^ii^	0.95	2.41	3.3532 (15)	173
C5—H5···O1	0.95	2.55	3.0484 (14)	113
C8—H8···O2	0.95	2.36	2.9049 (14)	116

Symmetry codes: (i) *x*, *y*, *z*−1; (ii) *x*, −*y*+1/2, *z*−1/2.
